# The Climate of Florida

**Published:** 1875-11

**Authors:** 


The Climate of Florida.—In response to your request that I should give you a short communication, for the columns of your paper, in regard to the climate of Florida, I would state very briefly my opinion of its advantages as a home for the invalid. The question of a winter climate, or, as it is called professionally, a "winter cure" for delicate persons, and especially those who are laboring under dieases of the chest, or who have a proclivity thereto, has occupied my attention for a considerable time, and after as careful a review of the whole subject as I have been able to make, I would state, that in my judgment there is no section of this continent at all comparable to East Florida, especially on the head waters of the St. John's river, for a winter residence in an equable and genial climate. I have no hesitation in asserting that no *cold* climate (notwithstanding the idea which has prevailed at the North with some professsional men in regard to the invigorating influences of the climate of Minnesota upon consumptives) can be favorable for the prevention and cure of lung diseases. The various reasons which might be adduced, independent of the actual facts of experience in a strictly professional paper, would not be appropriate to your columns, and I would, therefore, state only two radical objections to all cold climates for delicate persons in winter. 1. It is absolutely essential to the consumptive invalid that he should be able to spend the greater part of his time in the open air, and this can not be done in any cold climate without the greatest *discomfort*, the mere sensations and enjoyment of the individual having a material bearing upon the health of the invalid. 2. To be able to expose himself at all in a cold climate he must so burden himself with clothing and wrappings, to prevent the aggravation of his symptoms from the effects of cold, as to inflict a most serious injury upon his vital forces in ways well understood, viz: burdening his body by the constant carrying of excessive heavy clothing, and rendering himself much more liable to the effects of acute catarrh from the absolute necessity of constant changes in clothing, arising from the great difference in the temperature between the *low* out door temperature and the *high* indoor temperature, indispensable to the most ordinary comfort when not actively exercising. But I had no design whatever of discussing a subject that opens a field not designed in your request, and better suited to the pages of a medical journal. A late writer observes: "Florida, the southermost State of the Union, has a history and characteristics peculiar to herself, and distinctive features from the other Southern States, though an interest in common with all. Though the first portion of the United States occupied by Europeans, she is yet a comparative wilderness, and, though her eventful history has been written in Latin, French, Spanish, and English, and pamphlets and papers innumerable have been published in relation to her, yet to the world she is a romance, and practically an unknown province just entering upon a new career of development and progress." And in no particular is this more strikingly true than in regard to its climatic peculiarities and advantages, which become immensely enhanced in value by the most extraordinary and interesting scene now presented in the resettlement of the State by people, from not only every section of this continent, but from many parts of the Old World. Even the invalid, laboring under the apprehension of soon becoming a victim to that fell destroyer, consumption, is encouraged to make an effort for a new lease of life where all around him is so inviting. There is scarcely a day that he cannot spend in the open air, and if an actual settler spend his time in the light and interesting work connected with its semi-tropical fruit culture, and if a visitor with leisure at command, the innumerable lakes affording the most attractive and perfectly safe opportunities for pleasure-boating, and abounding with a great variety of fish, and the forest filled with game, light the fires of life anew, and give him a better opportunity of regaining his health than can probably be found elsewhere in the world. From my personal observations in Florida, strictly in regard to climatic advantages, I would recommend, as already stated, the section embracing, in general terms, the region bordering upon the headwaters of the St. John's, which also affords *one* of the best (if not the best) sections for the growth of semi-tropical fruit—an important item, at least to the invalid *settler*, and of no small consideration even to the mere *visitor*. To enlarge the area to somewhat greater limits, I would state that in my judgment the counties of Marion, Sumpter, Volusia and Orange afford probably the greatest climatic advantages for the winter, and of these I would select the county of Orange as superior to every other section of the State, lying, as it does, some thirty miles from the Atlantic ocean, and thus, while enjoying the benefit of its delightful breezes, at the same time being so remote as to escape the force of its storms, and, upon the other hand, being protected from the harsh winds of the northwest, and of the gulf by a distance of some sixty miles from the latter, and of some five hundred miles to the first mountain in the direction of the former. In conclusion of this hasty and desultory reply to your request, I would say that there are indications, of a decided character that Florida, and especially the portions indicated, beside continuing to be the permanent winter resort for all persons upon this continent desiring a mild and genial climate, either for health or pleasure, will also become one one of the most attractive winter resorts for the entire world.

